# Change in Number of OB/GYN Physicians Practicing Obstetrics After the *Dobbs* Decision

**DOI:** 10.1001/jamanetworkopen.2025.24893

**Published:** 2025-07-31

**Authors:** J. Edward McEachern, T. Allen Traylor, Deb Roman

**Affiliations:** 1 St Luke’s Health Plan, Boise, Idaho; 2 School of Public and Population Health, Boise State University, Boise, Idaho; 3 School of Health Sciences, Boise State University, Boise, Idaho; 4 Ada County Medical Society, Boise, Idaho

## Abstract

This cohort study evaluates changes in the number of obstetrics and gynecology physicians practicing in Idaho after the *Dobbs v Jackson Women’s Health Organization *Supreme Court decision.

## Introduction

In June 2022, the Supreme Court overturned *Roe v Wade* (*Dobbs v Jackson Women’s Health Organization* [*Dobbs*]^1^), activating Idaho’s trigger laws, making abortion or an “attempt to perform an abortion” for any reason other than the imminent death of the mother or in cases of rape or incest documented by a police report illegal. Penalties include license revocation, felony charges, minimum jail time, and fines (Idaho Code § 18-622, 2023^2^ and Idaho Code § 18-604, 2023^3^). This study assessed changes in the number of obstetrics and gynecology (OB/GYN) physicians practicing obstetrics in Idaho following implementation of these laws.

## Methods

Three cross-sectional censuses assessing the number of all OB/GYN physicians practicing obstetrics in Idaho were conducted in August 2022 (baseline), November 2023, and December 2024, following Strengthening the Reporting of Observational Studies in Epidemiology (STROBE) reporting guidelines for cohort studies under institutional review board approval from Boise State University. The need for informed consent was waived because data were deidentified. Data was compiled from publicly available credentialing files and sources, licensing boards, physician websites, Idaho’s American College of Obstetricians and Gynecologists registry, Idaho Medical Association database, local knowledge of the practices, and direct physician confirmation. The study tracked physicians who relocated, stopped practicing obstetrics, died, or retired, and added newly credentialed physicians. Locus tenens OB/GYN physicians and family medicine physicians practicing obstetrics were not included.

## Results

Between August 2022 and December 2024, repeated cross-sectional censuses revealed that Idaho lost 94 of the 268 OB/GYN physicians practicing obstetrics (35%), net new entrants to the state. Over the study period, 20 new OB/GYN physicians moved to Idaho, and 114 of the 268 baseline obstetricians (43%) stopped practicing obstetrics, left the state, closed their practices within the state, or retired. Notably, during the 15 months following the implementation of the post-*Dobbs* abortion laws in Idaho, 60 OB/GYN physicians stopped practicing obstetrics in the state (Figure). We identified the disposition of all 55 OB/GYN physicians who left obstetrics in Idaho in 2024 (Table). Of Idaho’s 44 counties, 151 of the state’s OB/GYN physicians practicing obstetrics (85%) are concentrated in the 7 most populated. In the remaining 37 counties, by the study’s conclusion, 23 OB/GYN practicing obstetricians served a population of 569 000 Idahoans.^4^

**Figure.**
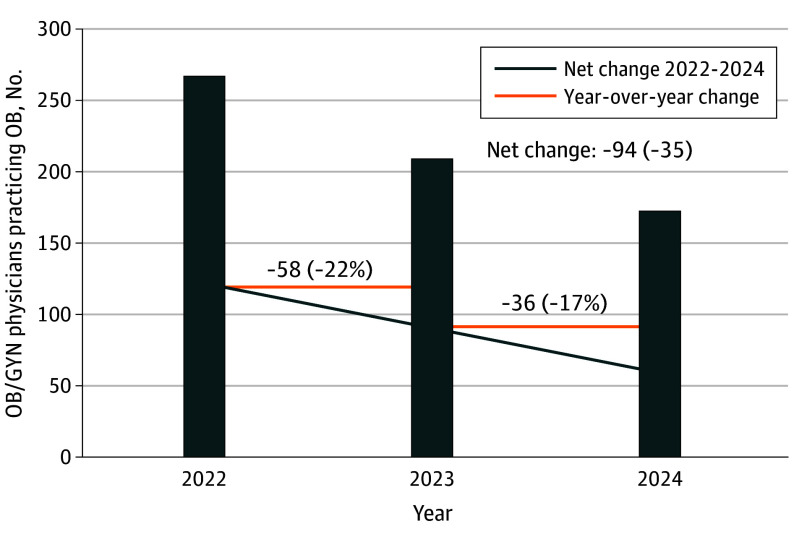
Change in Number of Obstetrics and Gynecology (OB/GYN) Physicians Practicing Obstetrics in Idaho Following the *Dobbs* Decision, 2022 to 2024

**Table.** Disposition of the 55 Obstetrics and Gynecology (OB/GYN) Physicians Who Left the Practice of Obstetrics in Idaho in 2024^a^

Disposition	No. (%)
Retired	12 (22)
GYN only	9 (16)
Left rural practice to consolidate practice in urban practice site	7 (13)
Move out of state^b^	23 (42)
In-state move	4 (7)

^a^ None of the OB/GYN physicians who left Idaho moved to states with abortion-restricted policies similar to Idaho. Twenty-two OB/GYN physicians moved from Idaho to contiguous states.

^b^ Out-of-state moves: 5 to Washington; 3 each to Minnesota, Nevada, Oregon, and Utah; 2 to Colorado; 1 each to Illinois, Maine, Montana, and New York.

## Discussion

Geographically, Idaho is the eleventh largest state in the US, with 2 million residents, including approximately 1 million women,^4^ many of whom have limited access to obstetricians or birthing centers. While this study was limited in size and only to Idaho, the loss of OB/GYN physicians practicing obstetrics, difficulty recruiting new obstetricians to the state, consolidation of practices in urban areas, and lack of an OB/GYN residency in Idaho may have implications for access to health care.

This study tracked all OB/GYNs practicing obstetrics in Idaho using multiple public sources and direct confirmation, providing detailed post-*Dobbs* practice data. The small sample size direct confirmation restricts replication in more populous regions and has limited generalizability. However, unlike studies that rely exclusively on administrative databases, this approach captured individual practice alterations that conventional credentialing data typically may not include.

A large decrease in the number of OB/GYN physicians practicing obstetrics was observed following changes in state-level abortion laws after *Dobbs*. Idaho has low numbers of OB/GYN physicians per capita,^5^ so a reduction in the workforce may pose a threat to health care access and broader community health.

## Notes

Supplement.
Data Sharing Statement

## References

[1] *Dobbs v. Jackson Women’s Health Organization*, 597 U.S. Ct. 2228. 2022.

[2] Code I. § 18-622 (2023). Abortion [State law]. 2023. Accessed June 23, 2025. https://legislature.idaho.gov/statutesrules/idstat/title18/t18ch6/sect18-622/

[3] Code I. § 18-604 (2023). Abortion and contraceptive definitions. 2023. Accessed June 23, 2025. https://legislature.idaho.gov/statutesrules/idstat/title18/t18ch6/sect18-604/

[4] U.S. Census Bureau. Idaho quick facts. 2024. Accessed June 23, 2025. https://www.census.gov/quickfacts/fact/table/ID

[5] Health Resources and Services Administration. National and regional projections of supply and demand for primary care practitioners: 2018-2030. U.S. Department of Health and Human Services. 2018. Accessed June 23, 2025. https://bhw.hrsa.gov/sites/default/files/bureau-health-workforce/data-research/projections-supply-demand-2018-2030.pdf

