# Supplementary material for: Change in Number of OB/GYN Physicians Practicing Obstetrics After the Dobbs Decision
Source: JAMA Netw Open. 2025 Jul 31;8(7):e2524893. doi: 10.1001/jamanetworkopen.2025.24893 (PMC12314713; doi:10.1001/jamanetworkopen.2025.24893)
Supplement: Supplement. — Data Sharing Statement [file jamanetwopen-e2524893-s001.pdf]

Data Sharing Statement

McEachern. Change in Number of OB/GYN Physicians Practicing ObstetricsAfter the Dobbs Decision. *JAMA Netw Open*. Published July 31, 2025.
doi:10.1001/jamanetworkopen.2025.24893

Data
Data available: Yes
Data types: Deidentified participant data
How to access data: request must be sent to allentraylor@boisestate.edu
When available: With publication

Supporting Documents
Document types: None

Additional Information
Who can access the data: researchers approved by an IRB
Types of analyses: to further advance the understanding of this issue
Mechanisms of data availability: with investigator support after approval of a proposal with a signed data access agreement
